# Ultrasensitive nano-gold labelled, duplex lateral flow immunochromatographic assay for early detection of sugarcane mosaic viruses

**DOI:** 10.1038/s41598-022-07950-6

**Published:** 2022-03-09

**Authors:** Raja Muthuramalingam Thangavelu, Nithya Kadirvel, Parameswari Balasubramaniam, Rasappa Viswanathan

**Affiliations:** 1 Plant Pathology Section, Division of Crop Protection, ICAR-Sugarcane Breeding Institute, Coimbatore, 641 007 India; 2 ICAR-National Bureau of Plant Genetic Resources, Regional Station, Hyderabad, 500030 India

**Keywords:** Biological techniques, Plant sciences

## Abstract

Sugarcane is one of the important food and bioenergy crops, cultivated all over the world except European continent. Like many other crops, sugarcane production and quality are hampered by various plant pathogens, among them viruses that infect systemically and cause severe impact to cane growth. The viruses are efficiently managed by their elimination through tissue culture combined with molecular diagnostics, which could detect virus titre often low at 10^–12^ g mL^−1^. To harmonize the virus diagnostics by molecular methods, we established a nanocatalysis-based high sensitive lateral flow immunochromatographic assay (LFIA) simultaneously to detect two major sugarcane viruses associated with mosaic disease in sugarcane. LFIA is known for poor sensitivity and stability with its signalling conjugates. However, we synthesized positively charged Cysteamine-gold nanoparticles and used them to prepare highly stable to sensitive immunoconjugates and as a colourimetric detection label. Further nanogold signal enhancement was performed on LFIA to obtain a high detection sensitivity, which is higher than the conventional immunoassays. The linear detection range of the nano-LIFA was 10^–6^ to 10^–9^ g mL^−1^, and with the signal enhancement, the LOD reached up to 10^–12^ g ml^−1^. This research paper provides relative merits and advancement on nano-LFIA for specific detection of sugarcane viruses in sugarcane for the first time.

## Introduction

Diagnosis of systemic viruses in sugarcane has been crucial to ensure safe and sustainable agriculture practices^1–4^. In particular, Sugarcane mosaic virus (SCMV) and Sugarcane streak mosaic virus (SCSMV) causing mosaic disease inflicts substantial economic losses for the farmers and associated sugar mills. Unlike other diseases, systemic infection of these viruses causes degeneration, i.e. loss of vigour; hence the growers harvest the canes but lose 30–50% of cane yield^5–8^. To date, point‐of‐care diagnosis and simple or grower‐friendly devices are lacking and specialized staffs are required to diagnose the diseases in the field. To circumvent, the researchers are actively involved in the development of in-field portable diagnostic devices. Lateral flow immunochromatographic assays (LFIAs) are the technology behind the simple, rapid and portable detection devices now popular in biomedicine, food, environment, and agriculture^9–13^. It is a solid-phase thin-layer chromatographic assay performed with the principles of antigen–antibody recognition reactions. LFIA is one of the widely used commercial point-of-care diagnostic methods for many infectious diseases^14^. However, some certain basic issues like poor and varying sensitivity and stability of conjugate labels significantly limit their usage in many applications, worldwide^15^.

Several approaches have been proposed to address the crisis and the most effective possibilities were found to be related to the signalling labels^14^. Advancement in LFIA has been achieved by using gold nanoparticles (GNPs) as protein-antibody labels^16,17^. Colloidal GNPs, specifically as colourimetric label synthesized by chemical reduction (Turkevich method) with the reducing agents like citrate, borohydride, CTAB (cetyl trimethyl ammonium bromide), ascorbic acid, carboxylic acids and biomolecules, etc.^18^. GNPs are a typical coloured (wine red) nanoparticle widely used in LFIAs because of their unique optical properties and significantly biocompatible physio-chemical properties, which provide high feasibility over conjugate probes (DNA, antibody, aptamer)^19–22^. GNPs based LFIA is now widely used to detect the economically important plant pathogens because of high detection sensitivity, user-friendly compared to conventional detection methods like ELISA, and dot-blot assays^23–25^. Moreover, GNP conjugates were successfully used to analyse varieties of bio-molecular binding interactions and the point-of-care detection of analytes, including highly important pathogens and food toxins^26–29^. On the other hand, researchers have bestowed some fascinating ingredients for improving the GNPs sensing phenomenon using silver and gold enhancement technology^30–34^. This enhancement method has been widely used in histochemical studies, where functional gold nanoparticles act as catalysts to reduce silver (Ag^+^) and gold (Au^+^) ions to metallic silver (Ag°) and gold (Au°). This autometallographic Ag and Au elemental deposition procedure enlarges the size of the core particles to tenfold larger and darkens the colour of the particles, becoming visible to the naked eye^30–34^.

The gold enlargement methods were well executed to detect economically important phytopathogen of Potato brown rot with the reported sensitivity of 3 × 10^4^ cells, which was 33-fold higher LOD than conventional nano-LFIA^35^. Similarly, nano-LFIA, after gold enlargement able to detect 230-fold reduced concentration (17 pg mL^−1^) of Potato virus X in the potato tuber samples^36^. Another destructive banana plant pathogen of Banana brack mosaic virus (BBrMV) detected up to 10 ng mL^−1^ by the conventional nano-LFIA^37^. Predominantly nano-LFIA assays operated Cit-AuNPs as conjugate and sensing labels. Our assay synthesized and used Cysteamine-AuNPs for high-affinity conjugation to antibodies that could improve the conventional LFIA sensitivity.

This research work discussed sensitivity and stability issues confronting in detail with the present LFIA. We developed duplex-nano-LFIA to detect two major sugarcane viruses simultaneously, namely Sugarcane mosaic virus (SCMV) and Sugarcane streak mosaic virus (SCSMV), under field conditions. Further, the detection signal improved to ultra-sensitive by gold enlargement method where the LOD was achieved at 10^–12^ g mL^−1^, which is equivalent to RT-PCR sensitivity. Hence, we are reporting for the first time that LFIA was developed for SCMV and SCSMV detection with a high sensitivity. As a conventional standard, dot blot immunoassay was performed to distinguish the relative sensitivity of nano-LFIA.

## Materials and methods

We used chemicals and materials essential for this research work from Sigma Aldrich and Merck Millipore with the highest purity. For the reagent preparation, nanoparticles dilution and other experimental works Ultrapure Milli-Q (Millipore-18 m Ω cm^−1^) water was used. All the glasswares (Borosil) needed for the nanoparticles processing was rinsed by Aqua regia (HCl: HNO_3_, 3:1) and thoroughly washed with Milli-Q water thrice, then oven-dried before use. LFIA kit from mdi Advanced Microdevices, Ambala, India was used in the assays. Antibodies specific to SCSMV was earlier produced from our laboratory^38^, and antibodies specific to SCMV were provided by Dr Stephen Winter, DSMZ, Germany. For dot blot assays and ELISA, alkaline phosphatase conjugated secondary IgG (Sigma-Aldrich, Bengaluru), NBT/BCIP stock solution (Roche diagnostics), pNPP (Nitrophenyl phosphate disodium salt from Sigma) were used.

### Virus sample extraction and purification

Healthy and virus infected sugarcane leaf samples (varieties Co 976, Co 7706 and CoC 671) collected from ICAR-SBI farm. The samples were confirmed for virus infection by RT-PCR assays with coat-protein gene specific primers^39,40^. Plant serum was prepared by homogenizing one gram of leaf tissue in the presence of liquid nitrogen with the sterilized ceramic mortar and pestle. Further the homogenate was pulverized with 10 or 20 volumes of extraction buffer [0.02 M phosphate buffer, 0.15 M NaCl, 0.02% Sodium azide, pH 7.4, 0.05% Tween20 and 2% PVP (40,000 MW)] and then filtered through two layers of mira or muslin cloth. Highly positive (isolate Co 7706) sample was used to purify the virus protein to use as a standard for this assay^41^. The purified virus protein quantified as 13.4 mg/ml by Nanodrop spectrophotometer (Thermo Scientific) at 260/280 nm.

### Synthesis of Cys-AuNPs

There are no standard protocols in the literature that enable direct preparation of cysteamine capped cationic nanoparticles for the specific purpose of antibody conjugation. Therefore, we adopted a protocol described elsewhere^42,43^ and modified the protocols to obtain stable cationic spherical gold nanoparticles with a narrow size distribution of 10–20 nm. Brought an aqueous solution of 40 ml HAuCl_4_ (1.4 mM) into a clean flask at room temperature and gradually added the aqueous solution of 400 μl (213 mM) of Cysteamine hydrochloride (C_2_H_7_NS) onto the solution under vigorous stirring. Twenty minutes later, a freshly prepared Sodium borohydride (NaBH_4_) of 40 μl (10 mM) was directly dropped into the reaction mixtures at the same stirring condition. The following 8–10 min was crucial to obtain the controlled synthesis of gold nanoparticles (AuNPs) in solution, also indicated by an intense wine red colour. The synthesized gold nanoparticles also rendered the striking red colour impression on the glass flask (anionic silicate ions), probably due to the attraction of cationic charged particles. Further, cysteamine stabilized gold particles were subjected to the standard characterization methods such as transmission electron microscopy (TEM), UV–Vis spectroscopy, zeta analysis for the size (d.m) and surface charge.

### Preparation of nanogold-immunoconjugates

The detection sensitivity of polyclonal antibody (pAbs) specific to the SCSMV and SCMV was initially evaluated by ELISA and dot blot assay (1:100–1:500 dilution) on different virus isolates infecting sugarcane. Before performing the conjugation, glycerol in the pAbs solutions was removed by Amicon 10 kDa centrifugal filter with the choice of PBS buffer. Both SCSMV and SCMV Cys-AuNP-conjugates were prepared separately. An amount of 50 µg mL antibody diluted in 0.01 M PBS containing 0.02% sodium azide was added directly to 1 ml of Cys-AuNPs (OD 530 = 1.8), and the suspensions were vortex mixed for one hour at 25˚C; after that, BSA and sucrose were added to a final concentration of 0.2% and 2% (w/v) respectively and again vortex mixed for 30 min. Meanwhile, the simple "salt challenge" method was performed by adding 1 M NaCl with nanogold-antibody conjugates to confirm their surface coverage, which must be protected from the salt-induced aggregation^23^. Subsequently, the spectra of the nanogold-labelled conjugates were recorded with UV–Vis absorbance spectroscopy.

### Stability of Cys-AuNPs

The stability of Cys-AuNPs studied in different thermal incubation (4, 10, 20, 30–35, 40, −20 °C and under light illumination) for 24 h, and the Cys-AuNPs-conjugates (SCSMV) stability studied from day 1 to 10, 20, 30 and 60 days at 4 °C. The samples were characteristically analysed through UV–Vis absorbance spectra with the AuNPs corresponding λmax and recorded data.

### Preparation of LFIA

The LFIA strip (refer graphical abstract) comprised of four components: a sample pad, a conjugate pad, Nitrocellulose membrane (NCM, pore size 8 μm), and an absorbent pad. All the membranes were sourced from a commercially available LFIA preparation kit. The kit provided different membrane types needs to be pre-selected based on the flow time and flow pattern. High sensitivity was obtained in smaller pore sized NCM (8 μm), but wicking time was comparatively more extended than the larger pore sized NCM. For faster results and high affinity, a larger pore size of NCM was the best choice. Finally, the assay performance might be vary for complexity of samples and conjugate label used. Before assembly of pads, conjugate pad pre-treated with PBS buffer (pH 7.4) containing 2% BSA, 2% PVP, 1% sucrose and 0.25% Tween 20 and allowed to completely dry at 37 °C for 2 h or RT for 24 h. This combination of buffer components was new and we tried especially for a thorough release of Cys-AuNPs-conjugates from the conjugate pad. Then, the desired volume of Cys-AuNPs-conjugates (OD 1 at 530 nm) was directly dispensed by micro pipetting onto a pre-treated conjugate pad (width of 6 mm) at a single instance. The conjugate pad was allowed to dry at RT for 4–5 h to prevent the humidity before assembling. Next, the nitrocellulose pad was used to immobilize antibodies in the test and control line. The blocking buffer (0.1% BSA in PBS) diluted capture antibody [(1.0 mg/mL) (SCSMV or SCMV)] and rabbit IgG-secondary antibody (1 mg/mL, 1:100, 200) loaded on the two printing channels in mdi membrane printing workstation and printed as test line (T) and control line (C) on the NC membrane (4 mm gap) at a medium speed, and then dried at 37 °C for four h. The sample pad is often treated with saline-sodium citrate buffer (SSC 4x) for human samples, however for plant samples no such pre-treatment is required since it was already filtered through mira cloth. Thus prepared conjugate pad adhered and then sample pad (half-width overlapped) on the top of antibody printed NC pad, after which attached the wicking pad at the bottom of NC pad. After all, assembled in the backing card, the plate was then cut into 4 mm wide strips by a strip cutter or manually by scissors. The prepared test strips were stored in a drying oven for a few minutes, then backed and sealed in the aluminium pouch with a desiccant.

### Duplex LFIA preparation

Two conjugation pads were prepared separately with SCSMV and SCMV nanogold-conjugates for duplex detection assay and placed together (overlapped) in a single strip. Conjugate labels were subjected to mild sonication before loading onto the pads. As mentioned earlier, two test lines were printed (0.5 cm gap) on the NC membrane with the SCSMV and SCMV diluted (0.5 mg/ml) antibodies.

### Optimization and detection of LFIA

Principally, the LFIA performance was demonstrated by adding a series of diluted (1 × PBS) purified virus proteins (SCSMV) [10 µg/ml to 10 pg/ml]. The intensity of the test line was then image captured to establish a standard calibration curve. For that, 30 µl of the purified virus protein sample was dispensed on a sample pad, and allowed to react with AuNP-immunoconjugates for one minute (pre-incubation), then 50 µl of 1 × PBS was added to the sample pad to flow the conjugate contents towards the wicking pad. Pre-incubation might help to improve the detection sensitivity as well. Generally, the visibility of the test line and control line is defined as a positive, and the absence of a visible test line define as negative. With the purified protein sample, the absence of signal was recorded as ultra-low signal. “The presence or absence of a control line respective to the assay is correct or false”. Similarly, the duplex assay was performed with the standard virus protein dilutions, and resulting images were captured for further analysis. Relative sensitivity of this LFIA was also determined through the standard Dot blot assay with SCSMV (1:500) antiserum (96 well Bio-Dot, Biorad) with alkaline phosphatase enzyme conjugates (1:2000) and NBT/BCIP substrates^44^. The output of colour intensity was image captured and measured to correlate with the enhanced nano-LFIA results.

### LFIA with citrate-AuNPs versus cysteamine-AuNPs

The comparative sensitivity of one-week-old LFIA strips imbedded with the conjugates of Citrate-AuNPs and Cysteamine-AuNPs was tested using two-fold diluted purified SCSMV, and the intensity of test and control line images were recorded. Citrate-AuNPs synthesized by standard procedure and antibodies were conjugated followed by the protocol described elsewhere^27,45^. The average size of the Cit-AuNP obtained was 30 nm in diameter, spherical in shape, and the surface charge was usual electronegative (data not shown). The size and shape of both AuNPs were much similar, and major variation occured in their surface properties. Significantly, the surface properties are the directing factor that controls the orientation of antibodies on the AuNPs. To verify this statement, more solid evidences were generated through the novel immunoassay testing of both conjugate-AuNPs with secondary IgG-conjugated with enzyme alkaline phosphatase (AP). The mechanism behind the test was how much AuNPs-conjugated-primary-IgG-(Fc region) remain accessible to the secondary-IgG (Fab region) (refer the illustration at Fig. a). Taken 1 ml of both Cit-AuNPs and Cys-AuNPs-immunoconjugates with the addition of 1:1000 diluted AP-conjugated-secondary-IgG, kept at room temperature for 1 h. Later, the solutions were centrifuged (12,000 rpm) to remove unbound secondary-IgG, and the pellets were redispersed to the final volume of 1 ml by Milli-Q. Further, dispersed the solutions (100 µl) into a 96well plate and each well was loaded with 100 µl of pNPP substrate to react with AP-conjugated-secondary-IgG. For this test, positive control was used with primary-IgG (1:1000) incubated with secondary-IgG-AP and water plus secondary-IgG-AP used as a negative control. Recorded the final colorimetric signal/OD value by using an ELISA reader at 405 nm (SpectraMax plus 384, Molecular devices, USA).

### Ultrasensitive detection by signal enhancement

All the standard dilutions that produced characteristic signals in LFIA were taken to the signal amplification procedure. Gold-enhancement solutions prepared as described elsewhere^46^. Briefly, solution 1: 2.5 mM HAuCl_4_ (gold chloride) was prepared at 10 mM MES buffer (pH 6), adjusted using 4 M NaOH solution. Solution 2: 1.027 M H_2_O_2_ prepared in 10 mM MES buffer 6. An equal volume of solutions 1 and 2 were mixed and used during the enhancement procedure. Before doing enhancement procedure, the NC pad was washed twice with washing buffer and dried to reduce background signals. For this assay, NC pad with test and control zones from the resulting strips separated by cutting and used to immerse in the instantly pooled gold enhancement solution 1 and 2 precisely for 2 min. The reaction was stopped immediately by dipping into DDH_2_O. Once the NC pad is immersed in the enhancement solution, the autocatalytic reaction is initiated to deposit the gold ions over GNPs, which increases the size of particles and gives dark blue colour in the test zones. Now, the test zones are visible to the naked eye and made easy for quantification by determining intensity level. The total events of gold nanoparticle hybridization detection to signal enhancement were captured in Scanning electron microscopy and presented here.

### In-field test

Nearly 10 sugarcane leaf samples were collected from different sugarcane cultivars (SCSMV—Co 06030, Co 86002, Co 96007, ISH 100, Co 14006, Co 6806; SCMV-CoBln 03174, CoS 94270, Madhurima, CoJaw 270), and plant serum was extracted to perform singleplex LFIA and recorded the results. Similarly, fresh field samples identified SCSMV and SCMV susceptible varieties (SCSMV & SCMV – Co 7706, CoA 96081, CoS 94270, CoC 671 and CoC 24) were collected and tested against the performance of the duplex assay and recorded the results. In addition, RT-PCR assays were performed to interpret the efficacy of nano-LFIA field tests (Table 1).

**Table 1:** Details of the sugarcane samples subjected to Nano-LFIA singleplex/Duplex assay and with the RT- PCR interpretation.

Sample nos	Cultivar/sample	Degree of symptoms	Results of nano-LFIA/Duplex assay		RT-PCR interpretation	
			SCSMV	SCMV	SCSMV	SCMV
1	Co 06030	++	+	−	+	–
2	Co 86002	+	+	−	+	−
3	Co 96007	+	+	−	+	−
4	ISH 100	+	+	−	+	−
5	Co 14006	+	+	−	+	−
6	Co 6806	+	+	−	+	−
7	CoBln 03174	+	−	+	−	+
8	CoS 94270	+	−	+	−	+
9	Madhurima	+	−	−	−	−
10	CoJaw 270	++	−	−	−	−
11	CoC 671	++	+	+	+	−
12	Co 7706	+	+	+	+	−
13	CoA 96081	+	+	+	+	−
14	CoS 94270	+	+	+	+	−
15	CoC 24	−	−	−	−	−

### Interpretation and distribution of data

All the data compared with a standard and controls were analyzed by the standard statistical procedures. The experiments was repeated wherever necessary to minimize random errors and achieve statistical significance: MS Excel and Origin 8 pro software are used to organize and analyse data.

### Research involving plants

Our experimental research with plants was carried out fully complying with our Institutional and International guidelines—the collection of samples, safe-handling and disposing performed within our Institute premises.

## Results and discussion

### Physiochemical characteristic features of Cys-AuNPs

**Figure 1:**
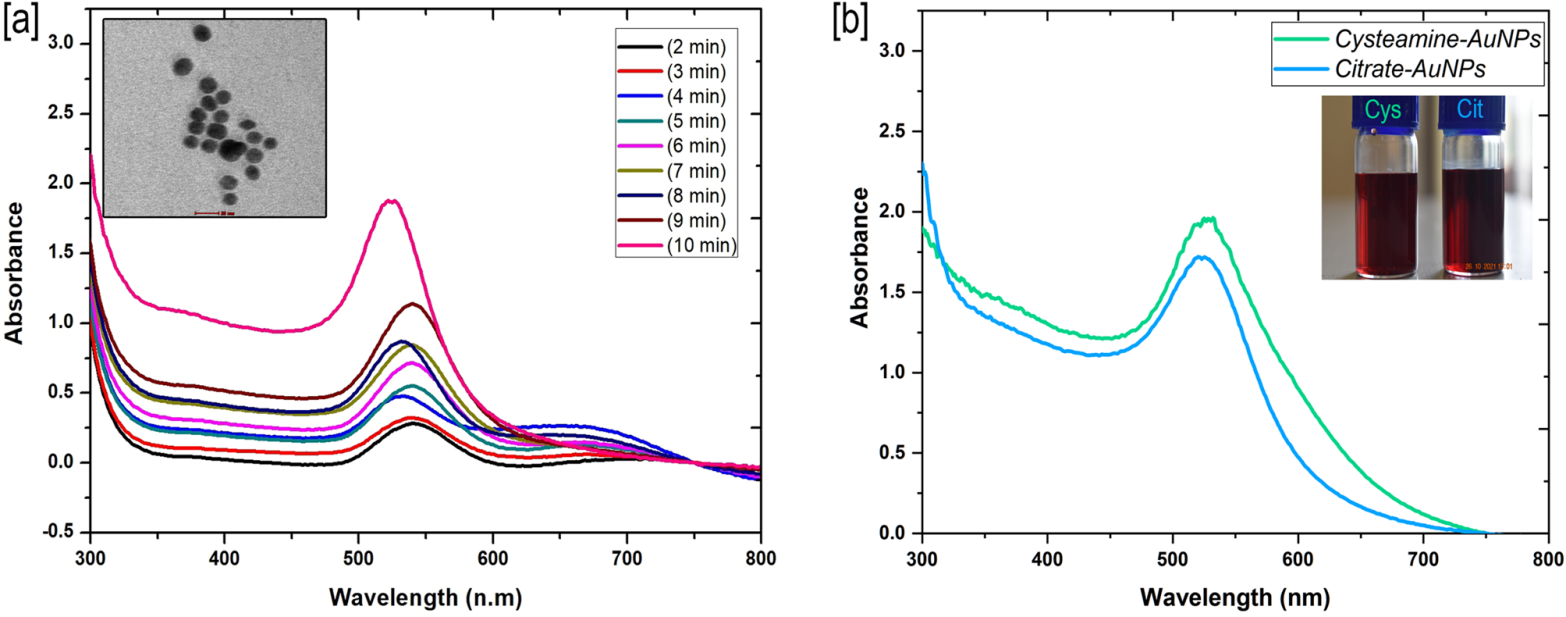
UV–visible absorbance spectral data corresponding to (**a**) Formation of highly stable colloidal Cys-AuNPs at different time intervals within ten minutes. (**b**) Comparative intensity of Cysteamine-AuNPs and Citrate-AuNPs, insets show synthesized colloidal gold nanoparticles.

**Figure 2:**
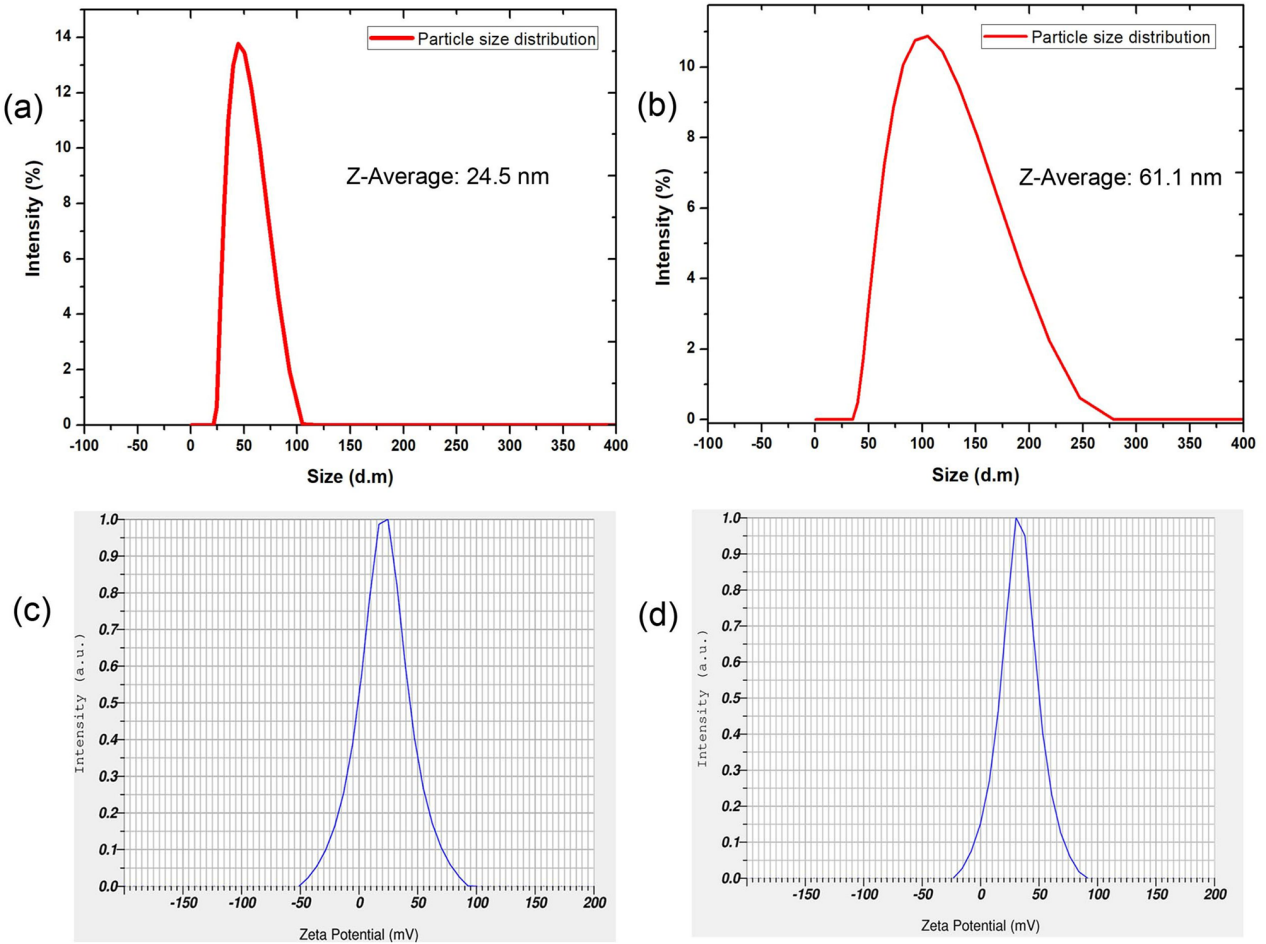
DLS particle size data corresponding to (**a**) Cys-AuNPs, (**b**) Cys-AuNPs-immunoconjugates; zeta potential surface charge measurement corresponding to (**c**) Cys-AuNPs, (**d**) Cys-AuNPs-immunoconjugates.

**Figure 3:**
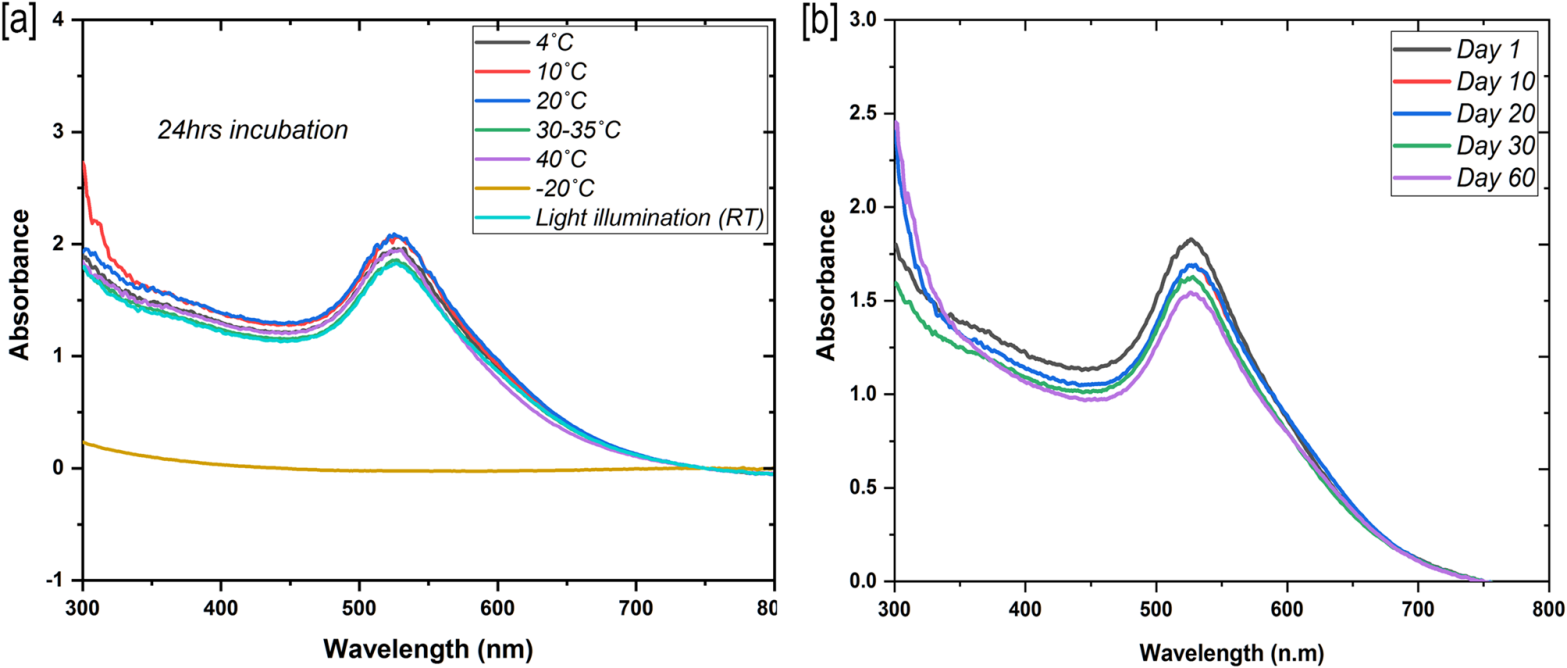
UV–visible absorbance spectra for stability test of Cys-AuNPs corresponding to (**a**) different thermal condition incubation for 24 h. (**b**) Incubation at 4 °C from Day 1 to Day 60.

The optical emission intensity (due to high monodispersity) of Cysteamine stabilized gold nanoparticles was comparatively brighter and more intense than Citrate capped gold nanoparticles that may help the enhanced colorimetric detection signal in the paper sensing platform (Fig. 1b). The colloidal solutions taken during particle synthesis (1–10 min) have shown the evidence of gradual reduction of auric ions (Au^3+^–A^0^) to nanoscale gold particles (AuNPs) under the analysis of UV–Vis absorption spectroscopy (Fig. 1a). The maximum (λmax) and strong absorbance peak of 520–540 nm was observed for the reaction solution taken at 10 min synthesis which is corresponding to the monodispersed colloidal gold nanoparticles (AuNPs) in the solution. It was seed mediated growth of AuNPs, principally initiated by thiol in the cysteamine and subsequently stabilized by the strong reducing agent of sodium borohydrate. Thiol is a highly attractive to metal ions involved in the nanogold seed synthesis further stabilized by borate ions. Average size of the Cys-AuNPs was determined as 24 nm (dm) through TEM and DLS and the shape was observed like spherical in the same image (Figs. 1a & inset, 2a). TEM image showed well dispersed nanoparticles by the cysteamine capping agent and borate stabilizing agent. We have had geometrically similar particles, consistently after many replications of synthesis that is a profound characteristic feature of Cys-AuNPs. The results of Zeta potential (mV) analysis for the Cys-AuNPs revealed that the electric potential and the surface charge of gold nanoparticle predicted as highly positive charged to the near value of + 30 mV in the graph and found some degree of measurable electronegativity in the same because of the borate (Fig. 2c). Zeta potential values of nanoparticles less than + 25 mV or greater than − 25 mV typically have low degrees of stability and dispersity in solution was reported in earlier study^47^. Consent with this statement, Cys-AuNPs (greater than 25 mV) in a storage glass bottles has shown good stability in months at 4ºC but not at the RT. Eventually, the behaviour of Cys-AuNPs at different thermal conditions incubated for 24 h showed greater stability at 4, 10, 20, 30–35 and 40 °C. As shown in Fig. 3a, no major distinct variation in the UV–Vis absorbance spectra was observed at the above-mentioned thermal conditions. Whereas when storage temperature was below 4 °C or freezing state (− 20 °C), particles were reverse aggregated by changing their colour to a bluish solution, and no absorbance peak was observed. Also, the solution exposed to light irradiation showed decreased absorbance maximum meant for the growth of particles in the solution due to the free-energy absorbance from photons.

### Cys-AuNPs as potential diagnostic-immunolabel

The functionalization of AuNPs with antibodies has been most important criteria and cumbersome process for successful immuno-sensing assays. Cysteamine (2-mercaptoethylamine) is a known organic compound consist of two functional groups such as amine and thiol which can be a potential coupling molecule to the antibodies^48^. However, carboxylate and sulfhydryl, consist in the antibodies are the most targeted and favourable conjugation sites for bioassays^49^. With the Cysteamine stabilized AuNPs, the presence of these two reactive binding sites were evidenced by the presence of positive charge (corresponding to amine) and modest negative charge (corresponding to thiol and borate) in the Zeta potential analysis was earlier discussed (Fig. 2c,d)^50^. Here, Cys-AuNPs might offer two strong interactions of ionic and covalent between the carboxyl tail and sulphur groups (through chemisorption) to the antibody, which are predominantly present in the Fc region (Fragment crystallizable region) and not in the antibody-binding site^51^. Combined ionic and covalent bonding energy of the two ensured specific and ideally oriented immobilization of antibodies on AuNPs. Therefore, the antigen binding (Fab) sites become well accessible for binding with their virus antigens. Hence, promisingly, the cysteamine stabilized AuNPs could offer combination of a strong coupling strategy (ionic and disulfide) for a stable conjugation of antibodies on them and this was also evidenced in the DLS measurement (Fig. 2b). This feature might help to improve the virus antigen detection capability and sensitivity when compared to other custom used AuNPs conjugation methods. Further, for the confirmation, we have performed Fourier transform infrared spectroscopy (FTIR) to analyze the bonding and surface characteristics between the cysteamine and Cys-AuNPs. The overlaid infrared spectrum taken (model Shimadzu FT/IR-6800typeA) with standard calibration and procedure showed deformed spectral peak obtained at the 2900–2700 cm^−1^ corresponding to B–H stretch (boranes), which attributed to a reduced state of sodium borohydride, the well-known strong reducing compound involved in the AuNPs synthesis (Suppl. Figure 1). There was no reference alone for sodium borohydride executed. The IR spectra of cysteamine (bottom), exhibited two main stretches of high absorption peak dedicated to primary (N–H) amine (1592 cm^−1^) and sulfhydryl (–SH) groups (470 cm^−1^) with standard frequencies. To the connection, in the upper IR spectra, the variation with low-intensity peaks at high frequencies observed in Cys-AuNPs shifted the wave number 1592 cm^−1^ to 1658 cm^−1^ and 470 cm^−1^ to 676 cm^−1^ attributed to open chain amino group and disulfides (S–S stretch). Generally, thiols participate in a thiol-disulfide exchange or coupling of two thiol groups by the oxidation results the disulfide bridge (Suppl. Figure 1)^52^. Hence, we concluded that cysteamine has given active sites by the interconversion (oxidation) of both a primary amine and thiol molecules in the AuNPs synthesis process. FTIR results have very well correlated with the results discussed in this section earlier. Further, we observed the overlaid peak of 3014–2943 cm^−1^ was the characteristic broad feature of C–H stretching region present in the cysteamine which might be part of stabilization of Cys-AuNPs along with the boranes^53^. Storage temperature is another factor that influences the lateral flow assay sensitivity which is absolutely associated with antibody conjugate labels^54^. The structural and chemical stability of antibodies based on hydrogen bonds which are more stable at low temperature. According to our analysis, cysteamine capping produced more compatible binding to the antibodies, thus resulted no aggregation of nanogold immunoconjugates upto 60 days at 4 °C (Fig. 3b). Moreover, the detection sensitivity of stored conjugates was similar compared with freshly prepared conjugates.

### Performance of LFIA

**Figure 4:**
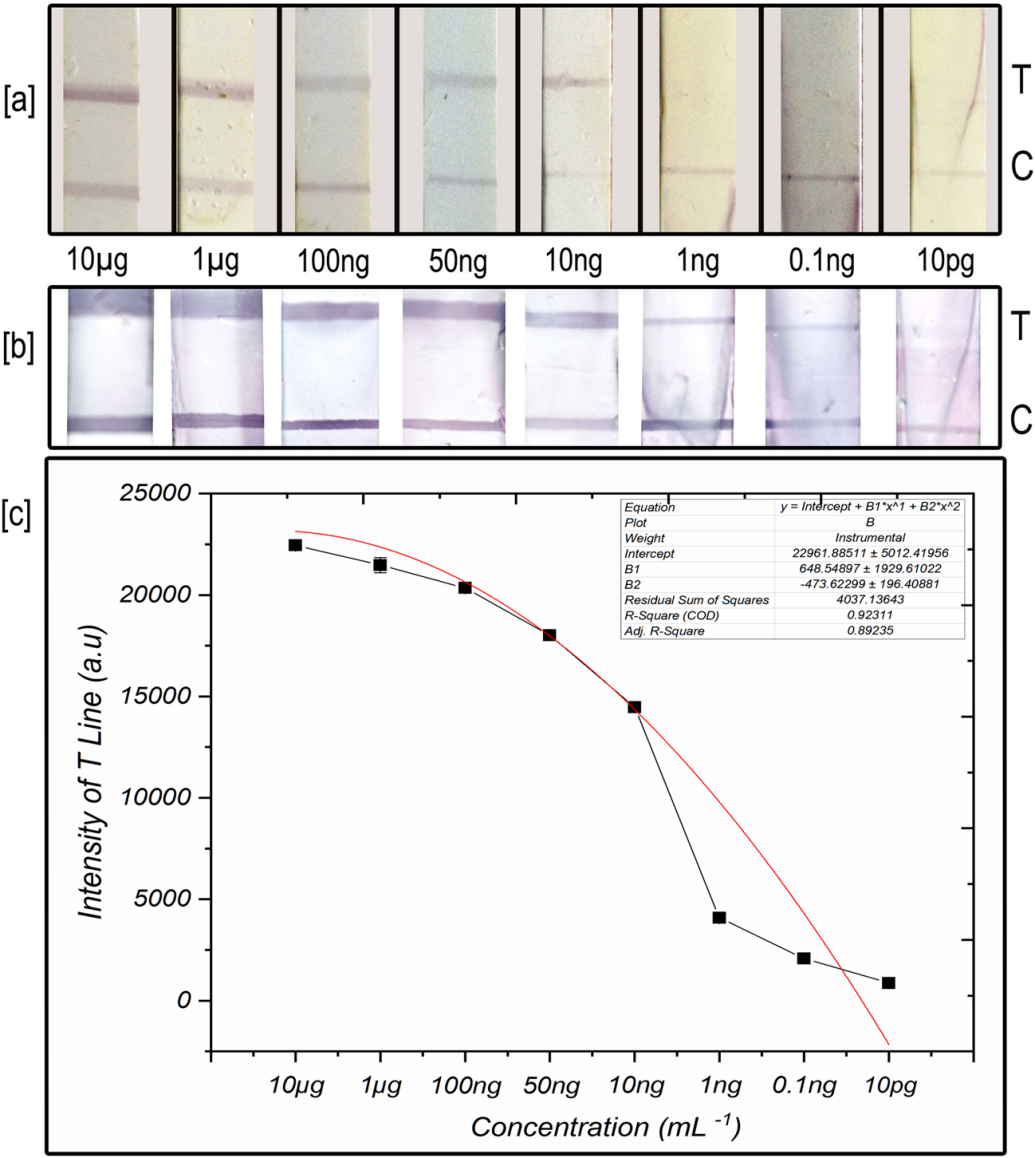
(**a**) The detection performance of nanogold-LFIA at various purified virus protein concentrations. (**b**) Strips after signal enhancement by gold enlargement method, (**c**) standard curve with polynomial fit generated from plate b corresponding to virus protein concentration Vs nanogold enhanced signal intensity.

**Figure 5:**
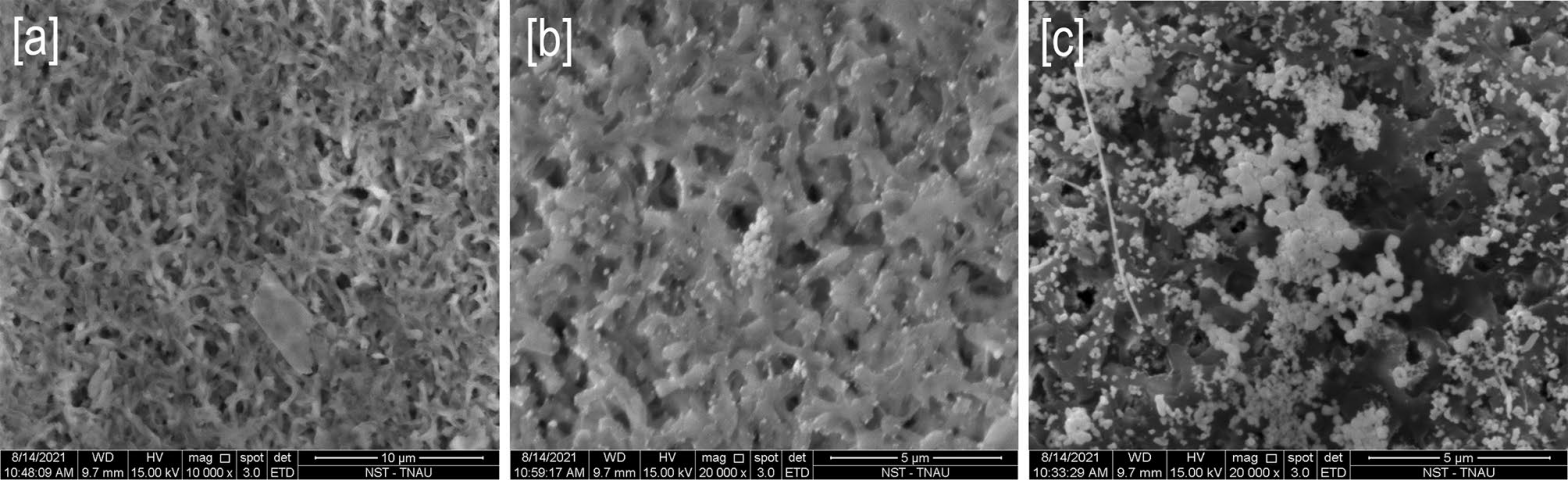
SEM microimages corresponding to (**a**) lateral flow nitrocellulose membrane before nanogold-immunoconjugates hybridization at 10 µm magnification, (**b**) after nanogold-immunoconjugates hybridization shown gold nanoparticles at 5 µm magnification, (**c**) after nanogold enlargement size increased to manifold at 5 µm magnification.

“LFIA”-based biosensing depends on the colourimetric signals originating from the gold nanoparticle hybridisation conjugated probes with target sugarcane viruses. LFIAs are easy to operate because the results can be acquired in a few minutes and are usually binary as positive or negative. In our LFIA, purified virus protein at the linear concentration of 10 µg to 10 ng showed a good detection sensitivity without the gold enhancement (Fig. 4a). The intensity of nanogold hybridized signal on the NCM was in high visibility to low visibility correlated with the linear concentration of virus protein tested. While the concentration close to 1 ng and below was not offered any visible signal in the T line. However, after placing the membranes into gold enhancement solution, the intensity of the T line signals was enhanced tenfold from the original signal, and the visibility improved at all the concentrations, including for the lowest concentration of 1 ng–10 pg (Fig. 4b). From the results of standard curve analysis, it was determined that the system of nanogold signal enhancement improved the sensitivity of sugarcane virus detection up to 10^–12^ g mL^−1^. The single-plex assay was effective as it responded well to all the sample concentrations. Control lines were visible in all the strips, and the intensity varied upon the hybridization event of the T line and with the flow rate of conjugate labels. From the signal enhancement results, a standard curve was generated with the polynomial fit, which showed a correlation among the various concentrations of tested virus protein, which could help to find the unknown concentration of virus, to be tested (Fig. 4c). SEM images taken with the test strips showed a series of differential occurrence of nanogold conjugates on the NCM before and after hybridization in the T line and then tenfold enlarged size particles after the application of gold enhancement solution (Fig. 5a–c). Moreover, the uniformity of enlarged size particles observed in the SEM images was clear evidence of controlled signal enhancement resulting from the 2 min precise incubation with the gold enhancement solution (Fig. 5c). Inappropriate incubation time with enhancement solution might cause the variation in the signal intensity from one set to another.

### Comparative sensitivity (Cit-AuNPs Vs Cys-AuNPs)

**Figure 6:**
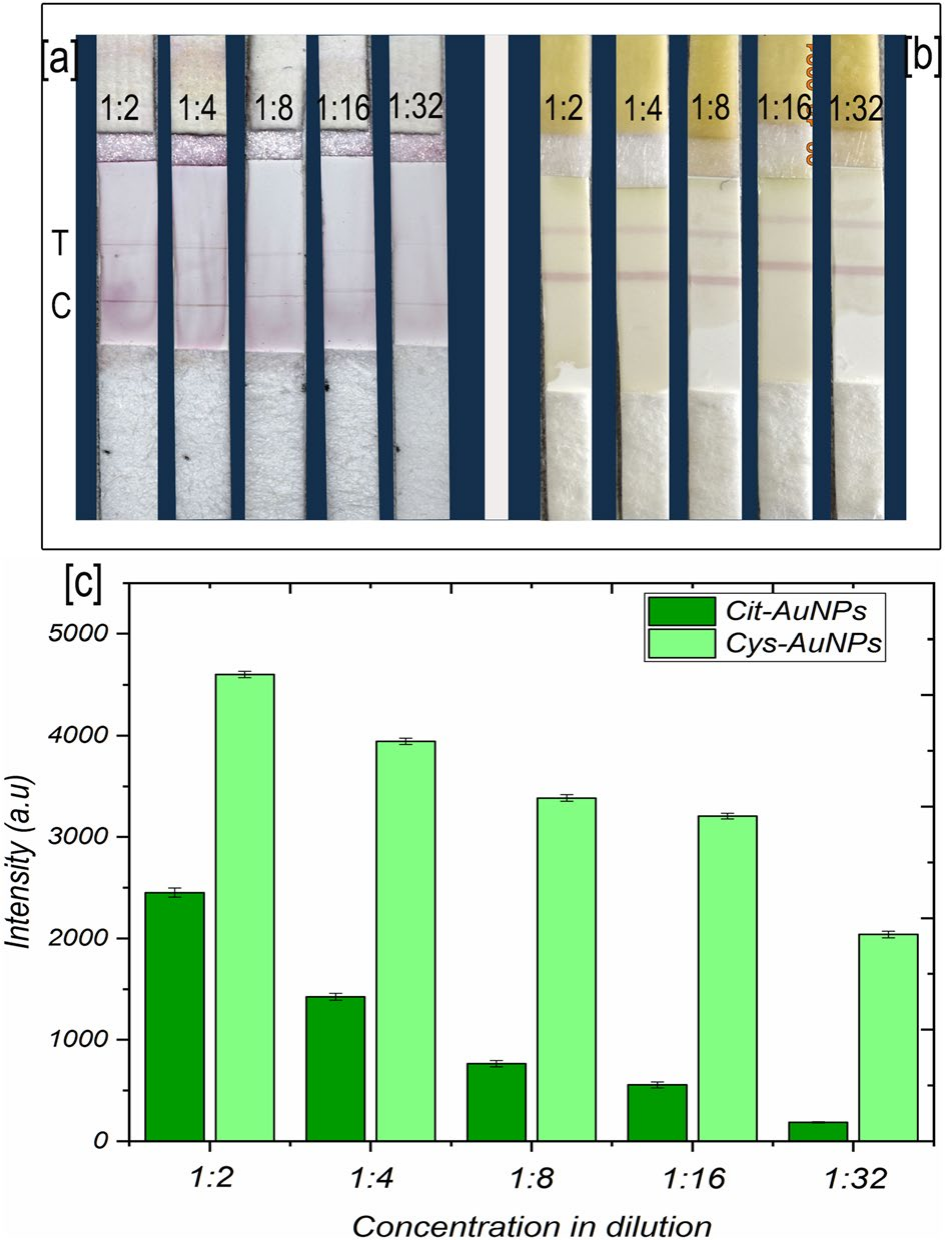
Comparative LFIA sensitivity with twofold diluted purified protein corresponding to (**a**) citrate-AuNPs-immunoconjugates, (**b**) Cys-AuNPs-immunoconjugates, and (**c**) histogram representation of T line signal intensity corresponding to plate (**a**) and (**b**). The error bars represent the standard deviation of three independents.

**Figure 7:**
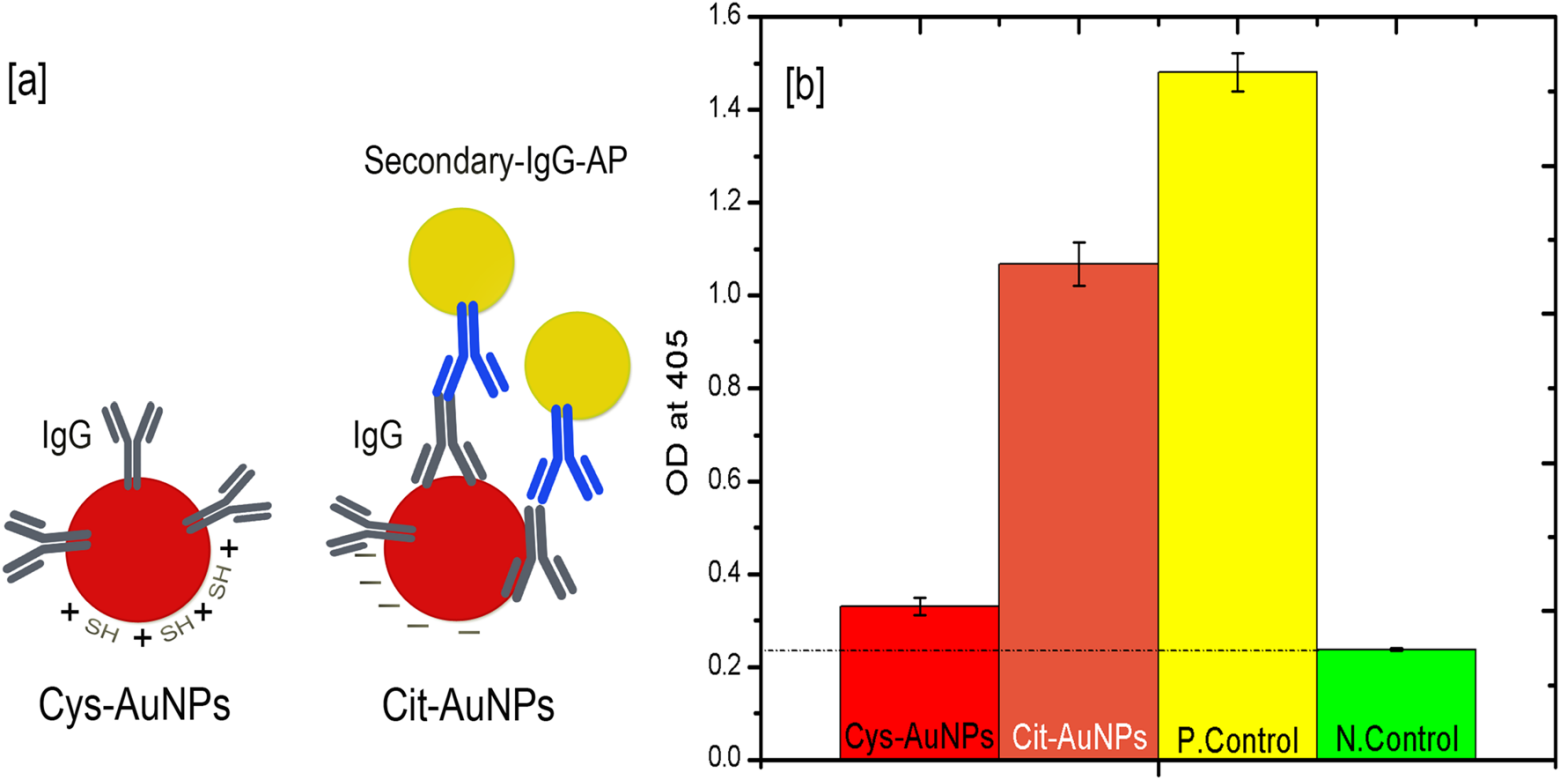
Study of antibody orientation via novel immunoassay: (**a**) illustration representing the possible antibody orientation to Cys-AuNPs and Cit-AuNPs along with the possibility of secondary-IgG-AP conjugates. (**b**) Colorimetric OD value obtained against the four samples tested that respective to Cys-AuNPs, Cit-AuNPs and two controls.

As briefly pointed out earlier, one-week-old citrate-AuNPs conjugates showed poor detection sensitivity in the lateral flow membrane at all the tested two-fold serially diluted purified SCSMV. In contrast, Cys-AuNPs conjugates in the lateral flow membrane showed improved sensitivity after one week of storage at 4 °C (Fig. 6a–c). Those images of LFIA indicate the differences of intensity in both T and C lines between the Cit-AuNPs and Cys-AuNPs. The calculated (Fig. 6c) detection sensitivity of Cys-AuNPs was tenfold higher than the Cit-AuNPs at the lowest virus concentration. Both gold nanoparticle geometries were similar; the surface chemistry alone played a critical role in proving longevity and sensitivity. The results of comparative sensitivity were apparent evidence for the difference between covalent conjugation of Cys-AuNPs and simple physisorption of Cit-AuNPs to the antibodies. This statement was demonstrated clearly with the proof from our antibody orientation study (Fig. 7a,b). From Fig. 7b, colourimetric OD values show the perfect distinction between the Cys-AuNPs and Cit-AuNPs-immunoconjugates reaction towards the secondary-IgG-AP. Compared to the positive control, a negligible amount of colourimetric signal was recorded for Cys-AuNPs-immunoconjugates. It denoted only the insignificant amount of the Fc region exposed to the solution and the remaining SCSMV antibodies oriented perfectly to Cys-AuNPs in an "end-on" position. Meanwhile, Cit-AuNPs showed almost fivefold higher than Cys-AuNPs and twofold lower than the positive control. It was evident that there was less possibility of perfect antibody orientation to Cit-AuNPs, which could be like Head/side-on position^55^. These results were the evidence of the stability of nanoparticle conjugates which were reinstated by the antibodies bonding and their steric hindrance to gold nanoparticles further improved the stability.

### Performance of duplex-LFIA assay

**Figure 8:**
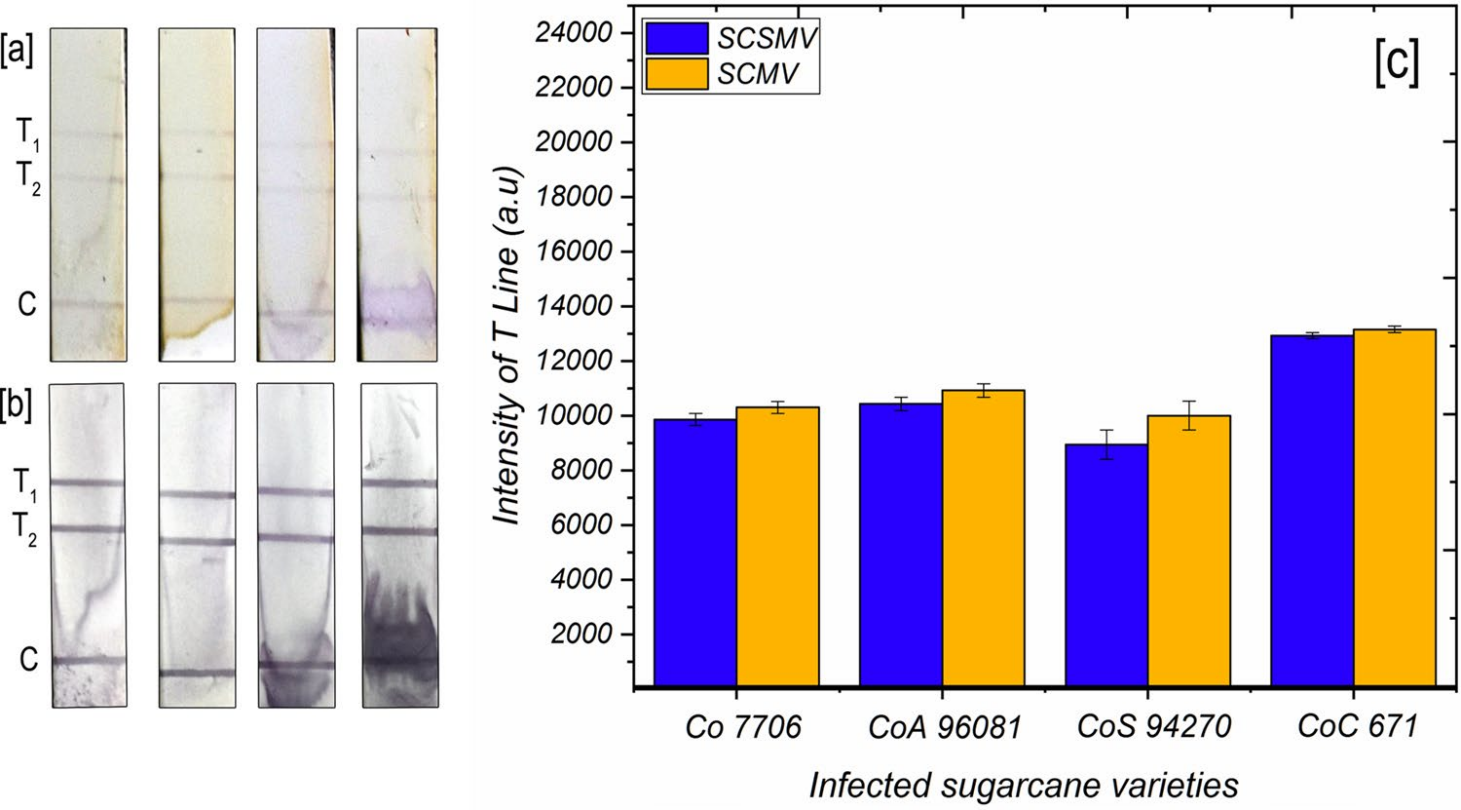
Duplex LFIA with four sugarcane samples infected with both SCSMV and SCMV Co 7706, CoA 96081, CoS 94270 and CoC 671 (**a**) duplex assay with two test lines corresponding to SCSMV and SCMV. (**b**) Signal enhancement after application of gold enhancement solution and (**c**) histogram representation of T_1_ and T_2_ line signal intensity corresponding to plate (**b**). The error bars represent the standard deviation of three independents.

The experimental results revealed that duplex assay could detect two important sugarcane viruses that predominantly coexisted in many important sugarcane cultivars. Duplex LFIA is never known for any plant virus detection. Also, embedding two conjugated reaction pads on one strip seems to be successful in our studies. We evaluated the efficacy of our duplex assay tested with four samples, and we observed slightly visible test lines of T1 & T2 in all the strips, corresponding to SCSMV and SCMV (Fig. 8a) (Table 1). Distribution of both viruses in one sample gives a lower intensity response in the two test lines as compared to the single-plex assay. Further, the poor detection signal was improved to tenfold after applying the gold enhancement solution, which facilitates clear visibility of both test lines and control lines (Fig. 8b). The enhanced signal intensity range was calculated and interpreted with the standard chart that showed the LOD of duplex assay between the ranges of 10 ng and 1 ng both viruses (Fig. 8c). This duplex assay also determined that the SCMV titre was a little higher than the SCSMV in the infected sample, as evidenced in the bar diagram (Fig. 8c).

### On-field diagnosis

Among the 10 samples tested in the nano-LFIA (Table 1), six samples were initially identified as positive for SCSMV with a visible low-intensity signal at T Lines (Suppl. Figure 2). The remaining four T lines corresponding to SCMV were subjected to gold enhancement application and found two samples were positive, and the last two were found negative. We found that the LFIA results 100% matched to RT-PCR analysis. The on-field diagnosis required 7-10 min to determine whether the samples were positive or negative with the ultrasensitive detection range of 10 ng to 1 pg. Nevertheless, the extraction of virus samples to be tested extended the overall testing time. Henceforth, we tried the juices extracted from virus-infected sugarcanes, and the results were found satisfactory with the help of a four-minute signal enhancement application. Two min pre-incubation of samples at conjugates pad found to give improved signal in the conventional LFIA. The specificity of our nano-LFIA tested with other sugarcane infecting viruses of *sugarcane yellow leaf virus* (ScYLV) and sugarcane bacilliform virus (SCBV) and found 99% accuracy in the specificity only for SCSMV and SCMV (Data not shown).

### Relative sensitivity

**Figure 9:**
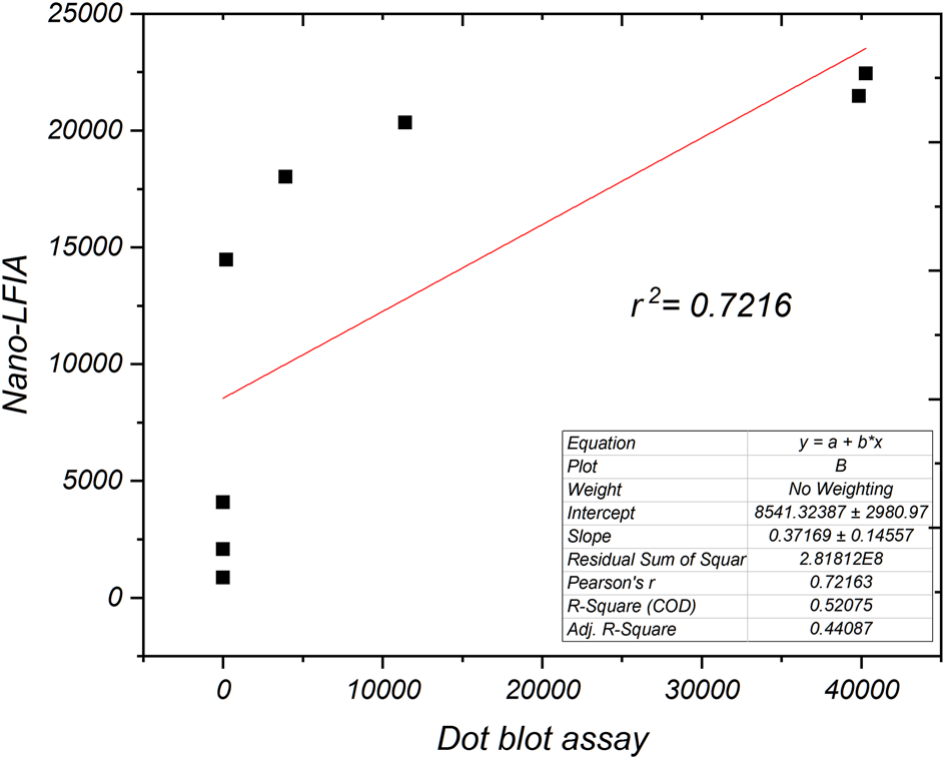
Pearson’s R^2^ correlation coefficient of detection sensitivity between nano-enhanced LFIA and dot blot assay with the purified SCSMV protein at 10 µg to 1 pg. The R^2^ value of 0.7216 indicates moderate positive correlation between two detection methods.

To estimate the relative sensitivity, statistical significance was generated from the results of dot-blot immunoassay with the variable purified virus concentration as mentioned earlier (Suppl. Figure 3). The estimated relative sensitivity between nano-enhanced-LFIA (Y-axis) and dot-blot assay (X-axis) found a moderate positive correlation, which means there is a tendency for high X variable scores to go with high Y variable scores (and vice versa) (Fig. 9). Briefly, nano-LFIA has shown an increased tendency in detecting variable virus concentrations. In contrast, the dot-blot assay showed poor sensitivity to the varying concentrations, which doesn’t statistically correlate with the nano-LFIA detection sensitivity. Hence it is assumed that nano-LFIA has a good linear sensitivity from the higher to lower virus titre than dot-blot assay.

### Summary

**Figure 10:**
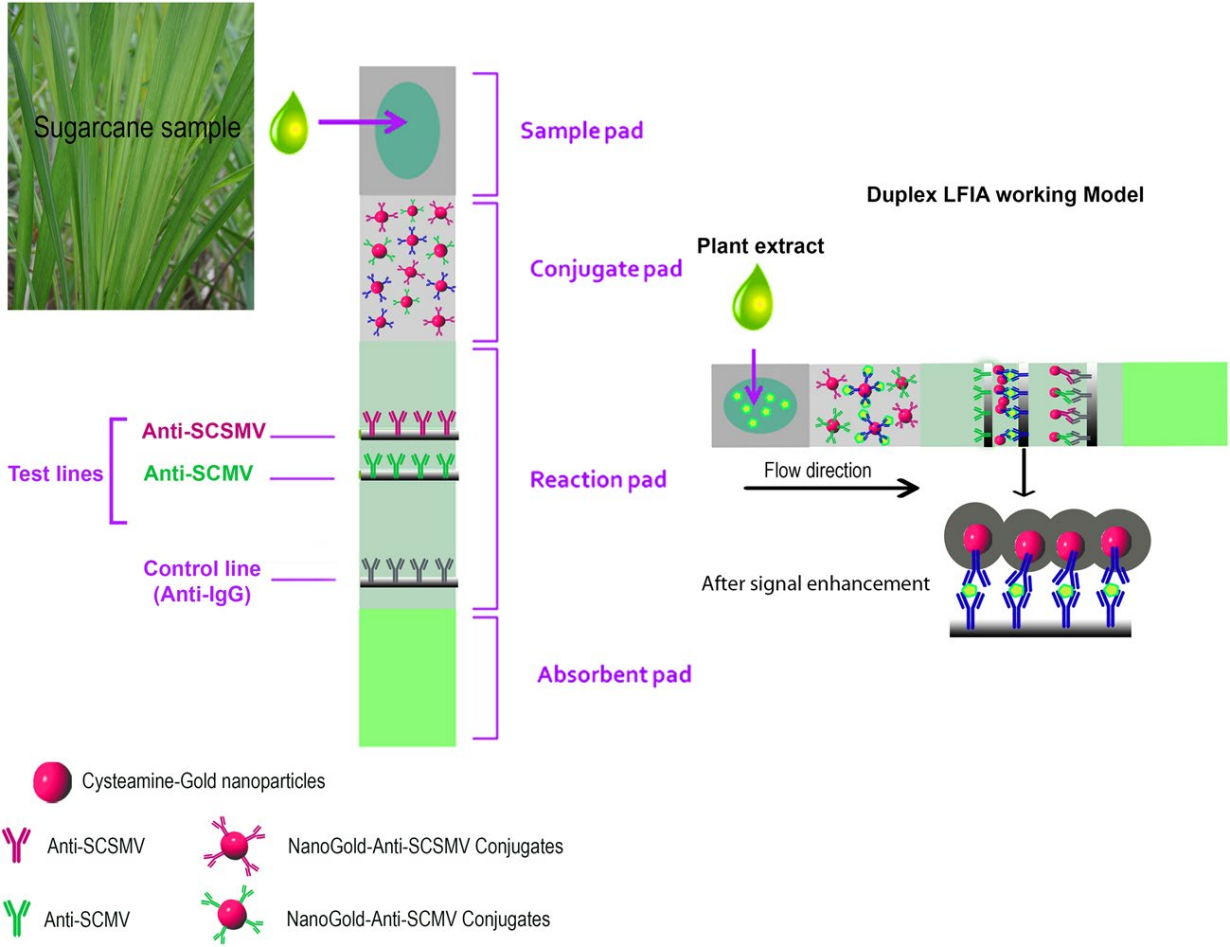
Graphical summary of ultrasensitive nano-gold labelled, LFIA for sugarcane viruses.

The low sensitivity and stability are the primary factors that prevent LFIA become an industry-standard diagnostics. Many researchers developed LFIA with increased sensitivity through much-distinguished modification such as various formats, different types of pads, different biorecognition and sensing labels than detection systems^56–60^. Likewise, we developed an LFIA with significant modification with conjugate tags that performed as a dynamic sensing label compared to a frequently used Cit-AuNPs. The signal enhancement system with gold nanoparticles is another excellent nanocatalytic method commonly used in LFIA and other gold nanoparticle-based sensors. Such a method is developed for the first time for sugarcane viruses with the linear detection sensitivity range of 10^–6^ to 10^–12^, almost equivalent to RT-PCR detection range. Moreover, increasing the incubation time of test lines at the enhancement solution may further improve the detection signal to 20-fold and more. However, it must be precisely applied to all the strips to be tested. This assay helps early and rapid diagnosis of SCSMV and SCMV in sugarcane seed materials where virus titre is often in the picomolar concentration. If LFIA is developed with monoclonal antibodies, the LOD will be on the scale of femtomole concentration. In addition, an approach of 2 min pre-incubation of the sample with conjugate labels in the conjugation pad could increase the sensitivity of the conventional LFIA^61^. Since, LFIA functioned in multipad components, the selection of pads are also the critical limiting factor for any successful LFIA development. Sensitive diagnostic assays are not applied for sugarcane diseases due to higher costs and a technical workforce with laboratory support. Hence, gold nanoparticle-based LFIA assay is economical and affordable for sugar industry and seed cane farmers in a county like India. It is also a practically feasible diagnostic assay for diagnosing sugarcane viruses and probably has a commercial value in the billion-dollar sugarcane industry (Fig. 10).

## Supplementary Information

Supplementary Information.

## Data Availability

All data analyzed during this study are included in this published article (and its Supplementary Information files).

## Abbreviations

LFIA: Lateral flow immunochromatographic assay
AuNPs & GNPs: Gold nanoparticles
SCSMV: Sugarcane streak mosaic virus
SCMV: Sugarcane mosaic virus
RT-PCR: Reverse transcriptase-Polymerase chain reaction
ELISA: Enzyme linked immunosorbent assay
Au: Gold
Ag: Silver
Cys: Cysteamine
Cit: Citrate NC: nitrocellulose
LOD: Limit of detection
TEM: Transmission electron microscopy
SEM: Scanning electron microscopy
DLS: Dynamic light scattering
FTIR: Fourier transform infrared spectroscopy
IgG: Immunoglobulin G
pNPP: para-Nitrophenyl phosphate
