# Supplementary material for: Ultrasensitive nano-gold labelled, duplex lateral flow immunochromatographic assay for early detection of sugarcane mosaic viruses
Source: Sci Rep. 2022 Mar 9;12:4144. doi: 10.1038/s41598-022-07950-6 (PMC8907228; doi:10.1038/s41598-022-07950-6)
Supplement: Supplementary file 1 — Supplementary Information. [file 41598_2022_7950_MOESM1_ESM.docx]

**Supplementary Information**

**Ultrasensitive nano-gold labelled, duplex lateral flow immunochromatographic assay for early detection of sugarcane mosaic viruses**

Raja Muthuramalingam Thangavelu^1^, Nithya Kadirvel^1^, Parameswari Balasubramaniam^2^, Rasappa Viswanathan^1^*,

1. Plant Pathology Section, Division of Crop Protection, ICAR-Sugarcane Breeding Institute, Coimbatore 641 007, India.
2. ICAR-National Bureau of Plant Genetic Resources, Regional Station, Hyderabad 500030, India

*Corresponding author: [rasaviswanathan@yahoo.co.in](mailto:rasaviswanathan@yahoo.co.in); ORCID- 0000-0002-7274-8144


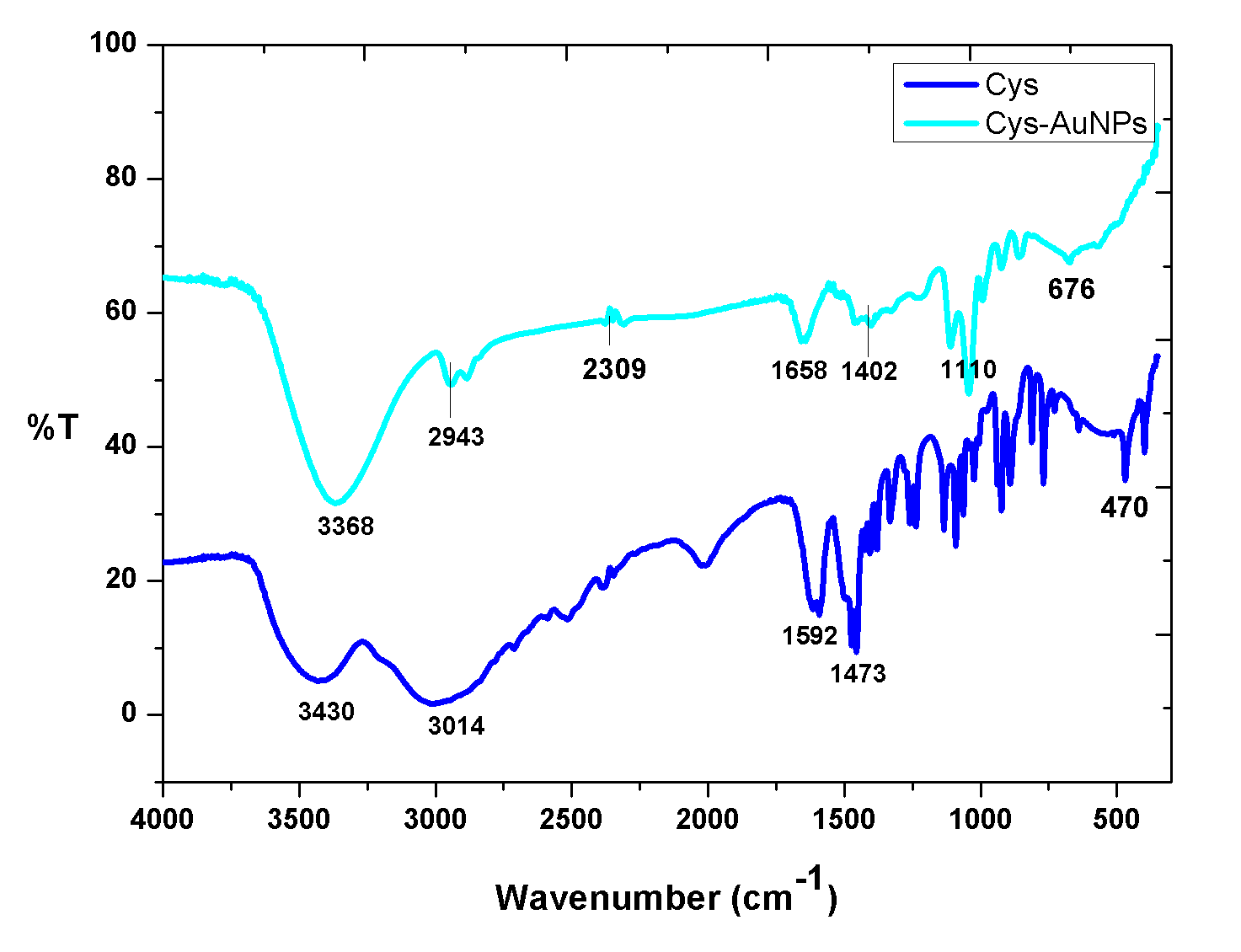


**S. Figure 1:** FITR overlaid spectra corresponding to pure cysteamine and cysteamine stabilized gold nanoparticles.


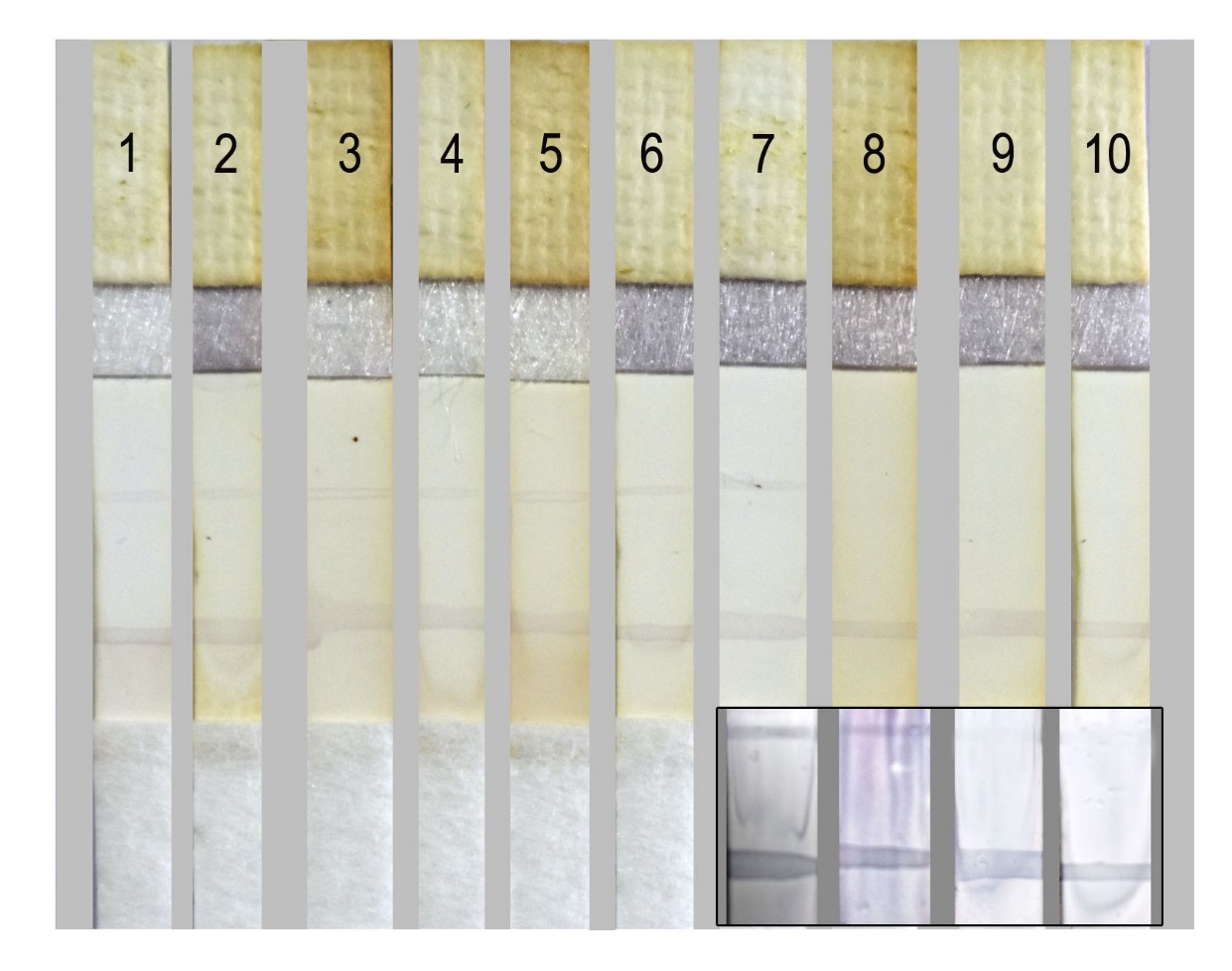


**S. Figure 2:** On-field lateral flow diagnosis for SCSMV and SCMV with 10 different samples (SCSMV – Co 06030, Co 86002, Co 96007, ISH 100, Co14006, Co 6806; SCMV- CoBln 03174, CoS 94270, Madhurima, CoJaw 270). Inset shown signal enhanced strips corresponding to low signal strips (7 & 8) that represented with low titre of virus. Result negative strips 9 &10.


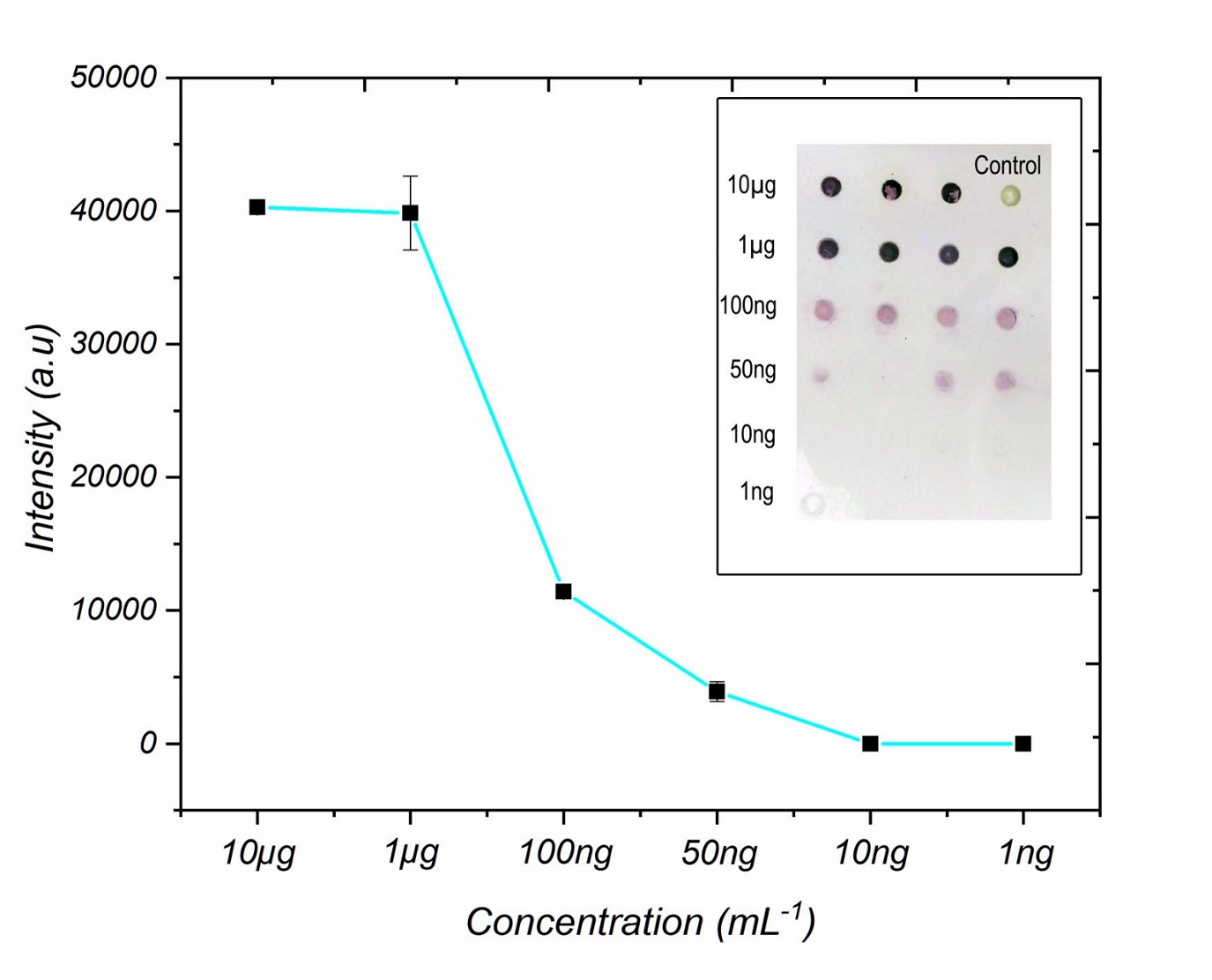


**S. Figure 3:** Dot-blot immunoassay performed with SCSMV antiserum/alkaline phosphatase secondary IgG/NBT-BCIP substrate produced dark to light indigo colour corresponding to the virus protein concentration. Graph representing the intensity against the concentration. The error bars represent the standard deviation of three independents.
